# Weight Gain and Metabolic Changes in Patients With First-Episode Psychosis or Early-Phase Schizophrenia Treated With Olanzapine: A Meta-Analysis

**DOI:** 10.1093/ijnp/pyad029

**Published:** 2023-06-16

**Authors:** Christoph U Correll, Mikkel Højlund, Christine Graham, Mark S Todtenkopf, David McDonnell, Adam Simmons

**Affiliations:** Department of Psychiatry, Zucker Hillside Hospital, Northwell Health, Glen Oaks, New York, USA; Department of Psychiatry and Molecular Medicine, Donald and Barbara Zucker School of Medicine at Hofstra/Northwell, Hempstead, New York, USA; Department of Child and Adolescent Psychiatry, Charité Universitätsmedizin, Berlin, Germany; Clinical Pharmacology, Pharmacy and Environmental Medicine, Department of Public Health, University of Southern Denmark, Odense, Denmark; Department of Psychiatry Aabenraa, Mental Health Services Region of Southern Denmark, Aabenraa, Denmark; Alkermes, Inc., Waltham, Massachusetts, USA; Alkermes, Inc., Waltham, Massachusetts, USA; Alkermes Pharma Ireland Ltd., Dublin, Ireland; Alkermes, Inc., Waltham, Massachusetts, USA

**Keywords:** Antipsychotic effect, body mass index, metabolic syndrome, psychosis

## Abstract

**Background:**

Patients with first-episode psychosis or early-phase schizophrenia are susceptible to olanzapine-associated weight gain and cardiometabolic dysregulation. This meta-analysis characterized weight and metabolic effects observed during olanzapine treatment in randomized clinical trials in this vulnerable patient population.

**Methods:**

PubMed, EMBASE, and Dialog were searched for randomized controlled trials (RCTs) reporting weight or cardiometabolic outcomes associated with olanzapine treatment in first-episode psychosis or early-phase schizophrenia. Random-effects meta-analysis and meta-regression were conducted using R v4.0.5.

**Results:**

Of 1203 records identified, 26 RCTs informed the analyses. The meta-analytic mean (95% CI) weight gain was 7.53 (6.42–8.63) kg in studies (n = 19) that reported weight gain with olanzapine treatment. Stratified by duration, the mean (95% CI) weight gain was significantly higher in studies >13 weeks in duration than in those lasting ≤13 weeks: 11.35 (10.05–12.65) vs 5.51 (4.73–6.28) kg, respectively. Despite between-study variability, increases from baseline in most glycemic and lipid parameters were generally small in studies of both ≤13 and >13 weeks. There were no correlations, however, between weight gain and metabolic parameter changes when stratified by study duration.

**Conclusions:**

In RCTs enrolling patients with first-episode psychosis or early-phase schizophrenia, olanzapine was consistently associated with weight gain that was greater in studies lasting >13 weeks compared with those of ≤13 weeks. Metabolic changes observed across studies suggest that RCTs may underestimate metabolic sequelae vs real-world treatment observations. Patients with first-episode psychosis or early-phase schizophrenia are vulnerable to olanzapine-associated weight gain; strategies minimizing olanzapine-associated weight gain should be carefully considered.

**Significance Statement**

Olanzapine is an effective antipsychotic medication for patients with schizophrenia or bipolar I disorder, but patients early in the course of illness may be more susceptible to olanzapine-associated weight gain and metabolic effects. To better understand the relationship between treatment duration, weight gain, and potential metabolic sequelae, we conducted a meta-analysis of randomized controlled trials to assess olanzapine-associated weight gain and metabolic outcomes in first-episode psychosis or early-phase schizophrenia. Olanzapine was consistently associated with weight gain that was greater in studies lasting longer than 13 weeks. There were changes across trial durations that may be indicative of metabolic sequelae; however, due to the limited duration of the studies, no correlations were observed between weight gain and these metabolic changes. Patients with first-episode psychosis or early-phase schizophrenia are vulnerable to weight gain while on olanzapine treatment, and strategies for minimizing ­olanzapine-associated weight gain should be carefully considered.

## INTRODUCTION

Antipsychotics used in the treatment of schizophrenia and bipolar I disorder are effective in reducing symptoms, but many are associated with weight gain and cardiometabolic side effects (De Hert et al., 2011; Leucht et al., 2013; Kishimoto et al., 2019; Pillinger et al., 2020). Weight gain associated with antipsychotic treatment is cited frequently by patients as bothersome and is a common reason for discontinuing medications, thus increasing the risk of symptom relapse and hospitalization (Law et al., 2008; Bessonova et al., 2020; Doane et al., 2020). Furthermore, weight gain is a risk factor for developing metabolic syndrome and cardiovascular disease, a leading cause of morbidity and mortality in patients with schizophrenia or bipolar I disorder (Vancampfort et al., 2015; Correll et al., 2017; Solmi et al., 2017).

Olanzapine, an atypical antipsychotic, is used for the treatment of schizophrenia or bipolar I disorder (Lieberman et al., 2005; Cipriani et al., 2011; Leucht et al., 2013; Yildiz et al., 2015). It is one of the most effective medications available for patients with multiepisode schizophrenia and has established efficacy in first-episode patients similar to that of other atypical antipsychotics (Zhu et al., 2017; Huhn et al., 2019). However, its clinical utility is limited owing to its propensity to cause weight gain and cardiometabolic dysfunction, including an increased risk of diabetes mellitus, dyslipidemia, and metabolic syndrome (Moisan et al., 2005; Meyer et al., 2008; Vancampfort et al., 2015). In olanza­pine clinical trials, weight gain and metabolic effects have been observed across diverse patient demographic populations; however, those who were antipsychotic naive or were otherwise early in the course of schizophrenia appeared to be particularly susceptible to these adverse olanzapine-associated treatment effects (Correll et al., 2009, 2014). Here, we sought to further explore these effects across clinical trials in patients who are early in their course of illness.

### Objective

This systematic review and meta-analysis examined olanza­pine data generated in the context of randomized controlled trials (RCTs) studying patients with first-episode psychosis or early-phase schizophrenia. In this analysis, the goals were to quantify weight gain (primary outcome) and cardiometabolic effects (secondary outcomes) associated with olanzapine treatment; to identify patient demographic, illness, and treatment factors that may modify these effects; and to evaluate the extent that olanzapine-associated weight gain may be related to changes in other cardiometabolic parameters.

## METHODS

### Protocol and Registration

Procedures for this meta-analysis followed the Preferred Reporting Items for Systematic Review and Meta-Analysis guidelines (Liberati et al., 2009). The Preferred Reporting Items for Systematic Review and Meta-Analysis checklist is included as supplementary Table 1. Analytic methods and study inclusion criteria were prespecified and documented.

### Information Sources and Search Strategy

A literature search was conducted through November 8, 2019, in the PubMed, EMBASE, and Dialog electronic databases using the following keywords, which were determined and agreed on by all authors: “olanzapine,” “schizophrenia,” “first episode,” and “early onset.” The search strategy for the PubMed database was “schizophrenia” [MeSH Terms] AND (“first episode” [All Fields] OR “early onset” [All Fields]) AND “olanzapine” [MeSH Terms] OR “olanzapine” [All Fields]. The search string for the EMBASE database was (“olanzapine”/exp AND ((“schizophrenia”/exp OR schizophrenia) AND (“first episode” OR “early onset”)) AND [EMBASE]/lim and [MEDLINE]/lim. The Dialog database was searched using schizophrenia AND (“first episode”) OR (“early onset”) AND olanzapine and (fdb(psycinfo)). No language or publication date restrictions were imposed.

### Eligibility Criteria

Eligible studies for this analysis were RCTs reporting on changes in body weight or cardiometabolic indices (ie, glucose or lipid metabolic parameters) observed during olanzapine treatment in patients with first-episode psychosis or early-phase schizophrenia, as defined in the individual respective publications. Studies analyzing multiple antipsychotics were included if the data were reported individually for olanzapine. Studies were excluded if they were not randomized, did not report on an outcome of interest, or did not include patients with first-episode psychosis or early-phase schizophrenia.

### Study Selection and Data Collection Process

The directed literature search and subsequent review of titles and abstracts for relevance, respectively, were conducted by medical staff (Barbara Zeman, PhD, and John H. Simmons, MD). Titles and abstracts of individual studies were screened to determine if the study met the criteria of a RCT. A.S. and C.G. confirmed relevance of the records identified to the research objective. If the relevance of a record was not clear by title and abstract review, the full-text article was obtained to determine if the report met eligibility criteria. In the event of uncertain eligibility, all authors convened to make the final determination. For RCTs comprising the final analysis set, data of interest were extracted directly from the full-text publications by A.S. then were confirmed by C.G. The extracted data were provided to M.H. and C.U.C. for meta-analysis and meta-regression.

### Data Items

The study characteristics that were extracted, when available, included the number of patients who were randomized to olanzapine and the number who completed the trial, the maximum duration of illness and maximum prior antipsychotic exposure allowed for entry into each respective study, the planned olanza­pine dose range, trial setting, and manner of trial blinding. Patient characteristics that were extracted, when available, included mean age of study participants, sex, race, baseline body mass index (BMI), underlying diagnosis, duration of illness, and prior antipsychotic exposure. Additionally, region (ie, China, Europe, India, international, or United States), target age demographic (children and adolescents, adults only, or mixed adolescent/adult population), and the mean olanzapine dose used in each respective study were captured.

### Risk of Bias in Individual Studies

All included RCTs were assessed for methodological quality by A.S. and C.G. using the updated Cochrane Collaboration’s tool for assessing risk of bias (Sterne et al., 2019) to extract data on study design and patient, illness, and treatment characteristics.

### Summary Measures and Methods of Analysis

The primary outcome of interest was mean change in weight between baseline and the respective individual study endpoints among patients with first-episode psychosis or early-phase schizophrenia who were randomized to olanzapine. Secondary outcomes of interest included mean changes in BMI, waist circumference, blood glucose, insulin levels, Homeostatic Model Assessment for Insulin Resistance (HOMA-IR), total cholesterol, triglycerides, low-density lipoprotein (LDL) cholesterol, and high-density lipoprotein (HDL) cholesterol between baseline and study endpoint.

### Statistical Analysis

#### Meta-Analyses

For the primary outcome of olanzapine-associated weight gain, we conducted a random-effects meta-analysis according to the DerSimonian and Laird method (DerSimonian et al., 1986) and used the metagen inverse variance function of R. The mean difference and 95% CIs were calculated as a measure of effect size, and between-study heterogeneity was assessed via the Higgins *I*^2^ statistic, with *I*^2^ > 50% indicating substantial heterogeneity. Data were summarized in a forest plot. We also performed prespecified subgroup analyses based on the following: region (China, Europe, India, international, or United States); prior antipsychotic exposure allowed in the RCT (≤4 or >4 weeks); and target age demographic (children and adolescents [<19 years of age], mixed adolescents and adults [≥13 years of age], or adults [≥18 years of age]). An additional subgroup analysis was performed to determine the effect of olanzapine treatment on weight gain in patients who were antipsychotic naive vs those who were not. Missing SDs for individual RCTs were imputed using Bracken’s approach for estimating a coefficient of variation from available SDs, taking the mean difference into account (Bracken, 1992). To assess the methodological quality of the included RCTs on the primary outcome of olanzapine-associated weight gain, we conducted a sensitivity analysis including only studies with a low risk of bias.

Secondary outcomes were also subjected to random-effects meta-analysis, as described above. Laboratory values for glucose and lipids (triglycerides; total, LDL, and HDL cholesterol) were converted to mg/dL before analysis. Corresponding SDs that could not be converted were imputed as described above (Bracken, 1992). Changes for each variable were based on deltas reported in the reviewed RCTs. If only baseline and endpoint mean values were reported, the changes could be calculated if at least 85% of patients remained on trial at endpoint.

All analyses were conducted with R v4.0.5 (R Foundation, 2002), and *P* < .05 was considered statistically significant.

#### Meta-Regression

To evaluate the influence of study and patient characteristics on the heterogeneity of olanzapine-associated weight gain and cardiometabolic outcomes, we conducted a mixed-effects meta-regression analysis using the metareg function of R to search for moderators in the identified RCTs. The prespecified outcomes of interest were weight gain, BMI, blood glucose, insulin levels, HOMA-IR, total cholesterol, triglycerides, LDL cholesterol, and HDL cholesterol. Covariates in the analysis included RCT duration (for studies lasting ≤13 weeks only), mean olanzapine dose, completion rate, mean patient age, and the percentage of male patients enrolled. Race was not included as a covariate because many studies were conducted in China or Europe; thus, by inference, the majority of enrolled patients across the analyzed studies were Asian or White, but specific data on race were missing in most studies. Regression coefficients and 95% CIs were calculated, and linear relationships between the covariates and the outcome variables of interest were considered statistically significant at *P* <.05. Correlations between weight gain and metabolic parameters were analyzed using simple linear regressions and assessed via regression coefficients and coefficients of determination (ie, R^2^).

#### Risk of Bias Across Studies

Funnel plots were inspected visually for asymmetry. In the case of asymmetry and at least 10 studies reporting an outcome, regression tests (Egger et al., 1997) and the trim-and-fill method (Duval et al., 2000) were used to evaluate the potential presence of publication bias and study heterogeneity.

## RESULTS

### Study Characteristics

**Figure 1.**
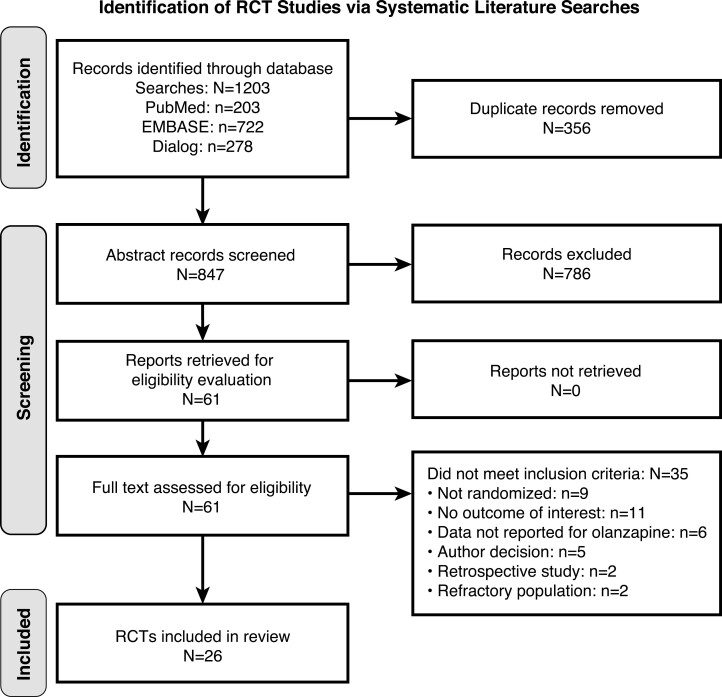
Flowchart of included and excluded studies. RCT, randomized controlled trial.

The literature search yielded 1203 records across the 3 databases (PubMed, n = 203; EMBASE, n = 722; and Dialog, n = 278; Figure 1). After the removal of 356 duplicates, 847 unique records remained. Title and abstract review resulted in 786 records being discarded for not meeting the eligibility criteria (eg, if they did not report results from a RCT, did not include the specific populations or outcomes of interest, or did not report olanzapine-specific data), leaving 61 potentially eligible reports. After full-text review of these 61 articles, 35 reports were excluded as irrelevant. The final data set included 26 RCTs that met eligibility criteria (Figure 1).

The identified studies reporting on olanzapine-associated weight gain or metabolic outcomes were categorized into 2 dichotomous groups, based on study duration, as ≤13 or >13 weeks. Three of the included studies reported data outcomes from both time frames (ie, ≤13 and >13 weeks) (Zipursky et al., 2005; McEvoy et al., 2007; Patel et al., 2009). Therefore, 19 data sets provided information from studies ≤13 weeks (Sanger et al., 1999; Poyurovsky et al., 2002; Lieberman et al., 2003; Zipursky et al., 2005; Wu et al., 2006; McEvoy et al., 2007; Perez-Iglesias et al., 2007; Wu et al., 2007; Saddichha et al., 2008a, 2008b, 2008c; Sikich et al., 2008; Patel et al., 2009; Li et al., 2012; Ou et al., 2013; Zhang et al., 2014; Huang et al., 2018; Kahn et al., 2018; Cheng et al., 2019). Of these studies, 13 were conducted with patients with first-episode schizophrenia (Poyurovsky et al., 2002; Wu et al., 2006, 2007; Saddichha et al., 2008a, 2008b, 2008c; Sikich et al., 2008; Li et al., 2012; Ou et al., 2013; Zhang et al., 2014; Huang et al., 2018; Kahn et al., 2018; Cheng et al., 2019). The remaining 6 reports included patients with first-episode psychosis (Sanger et al., 1999; Lieberman et al., 2003; Zipursky et al., 2005; McEvoy et al., 2007; Perez-Iglesias et al., 2007; Patel et al., 2009). One of these included studies evaluated the potential association between olanzapine-related weight gain and symptom improvement and found no correlation between these variables (Poyurovsky et al., 2002).

Weight or metabolic data were provided in 10 studies longer than 13 weeks (Zipursky et al., 2005; Green et al., 2006; McEvoy et al., 2007; Kahn et al., 2008; Perez-Iglesias et al., 2008; Arango et al., 2009; Patel et al., 2009; Perez-Iglesias et al., 2009; Findling et al., 2010; San et al., 2012). Of these 10 studies, 2 enrolled patients with first-episode schizophrenia (Kahn et al., 2008; Findling et al., 2010), and the remaining 8 studies enrolled patients with first-episode psychosis (Zipursky et al., 2005; Green et al., 2006; McEvoy et al., 2007; Perez-Iglesias et al., 2008; Arango et al., 2009; Patel et al., 2009; Perez-Iglesias et al., 2009; San et al., 2012). Two studies in this group analyzed the relationship between olanzapine-associated weight gain and clinical efficacy (Zipursky et al., 2005; Patel et al., 2009). Despite a 2018 systematic review of 31 independent studies (3 double-blind RCTs, 12 prospective studies, 13 post hoc analyses, and 6 chart reviews) that found a correlation between weight gain and therapeutic benefit in 7/9 (78%) olanzapine studies included there (Raben et al., 2018), only 1 RCT in the present analysis reported such a correlation. In that 1 study, a small, transient correlation of efficacy with weight gain was observed over the first 6 weeks of olanzapine dose titration but was not evident for the remainder of the 2-year study (Zipursky et al., 2005). Study and patient characteristics from the RCTs informing the analysis are reported in Table 1.

**Table 1.** Study Characteristics

First author, year/related study, year	Duration, wk	Blinding/setting	Diseases (max illness duration/max prior AP exposure)	Age category, y	Region (country)	No. randomized/No. completed (% randomized)	Mean OLZ dose, mg (SD)/planned dose range, mg/d	Mean age (SD), y	Male/ female, %	White/Black/Other, %	Mean illness duration, y (SD)/mean AP exposure, mo (SD)	Mean BMI at baseline, kg/m^2^ (SD)
Duration: ≤13 wk												
Cheng, 2019	8	Open-label/combined	First-episode schizophrenia (≤3 y/<12 wk)	Adults, 18–45	China (China)	168/135 (80)	16.0 (4.9)/10-25	24.6 (7.8)	51/49	0/0/100	NR/NR	NR
Huang, 2018	13	Single-blind/unknown	First-episode schizophrenia (<2 y/drug naive)	Adolescents/adults, 13–45	China (China)	29/25 (86)	17.8 (3.6)/NR	23.8 (5.9)	69/31	0/0/100	0.66 (0.72)/NR	20.4 (3.1)
Kahn, 2018	6	Double-blind/combined	First-episode schizophrenia (<2 y/<6 wk [+4 wk AMI in phase 1])	Adults, 18–40	Europe^a^	46/39 (85)	15.6 (6.5)/5-20	24.6 (5.5)	74/26	93/NA/7	0.6 (0.55)/NR	NR
Li, 2012	6	Open-label/mixed	First-episode schizophrenia (NS/NS)	Adults, 18–60	China (China)	40/40 (100)	19.1 (1.9)/5-20	23.6 (4.9)	70/30	0/0/100	0.59 (0.21)/NR	20.3 (2.9)
Lieberman, 2003/Zipursky, 2005	12	Double-blind/mixed	First-episode psychosis (<5 y/<16 wk)	Adolescents/adults, 16–40	International^b^	131/88 (67)	9.1 (NR)/5-10/20	23.5 (4.6)	79/21	51/37/12	NR/1.1 (1.4)	23.7 (4.9)
McEvoy, 2007/Patel, 2009	12	Double-blind/mixed	First-episode psychosis (<5 y/<16 wk)	Adolescents/adults, 16–40	USA (USA)	133/85 (64)	11.7 (5.3)/2.5-20	24.7 (5.8)	76/24	46/46/8	0.92 (1.1)/1.7 (2.2)	25.8 (6.2)
Ou, 2013	6	Open-label/mixed	First-episode schizophrenia (<1 y/<2 wk)	Adults, 18–45	China (China)	130/111 (85)	19.0 (2.3)/5-20	27.7 (8.0)	57/43	0/0/100	8.4 (4.4)/NR	20.7 (2.8)
Perez-Iglesias, 2007	12	Open-label/combined	First-episode psychosis (1st episode/drug naive)	Adolescents/adults, 15–50	Europe (Spain)	43/41 (95)	14.6 (3.5)/5-20	28.5 (6.5)	61/39	NR	1.2 (2.8)/NR	22.9 (3.0)
Poyurovsky, 2002	8	Double-blind/inpatient	First-episode schizophrenia (1st episode/ <4 wk)	Adults,^e^ NS	Europe (Israel)	15/13 (87)	10.0 (NR)/10	26.1 (7.9)	70/30	NR	2.2 (2.3)/NR	21.1 (3.3)
Saddichha, 2008a/Saddichha, 2008b/Saddichha, 2008c	6	Double-blind/inpatient	First-episode schizophrenia (NS/drug naive)	Adults,^e^ NS	India (India)	35/NR	16.5 (4.6)/NS	NR	51/49	0/0/100	NR/NR	NR
Sanger, 1999	6	Double-blind/inpatient	First-episode psychosis (<5 y/NS)	Adults,^e^ NS	International^c^	59/43 (73)	11.6 (5.9)/5-20	29.0 (7.8)	68/32	80/10/10	NR/NR	NR
Sikich, 2008	8	Double-blind/unknown	Early-onset schizophrenia spectrum disorder (NS/NS)	Children/adolescents, 8–19	USA (USA)	35/17 (49)	11.4 (5.0)/2.5-20	NR	71/29	60/34/6	NR/NR	23.5 (4.5)
Wu, 2006/Wu, 2007	8	Open-label/inpatient	First-episode schizophrenia (1st episode/drug naive)	Adults, 18–45	China (China)	24/24 (100)	13.7 (1.6)/10-20	34.2 (10.3)	58/42	0/0/100	0.16 (0.06)/NR	20.7 (0.3)
Zhang, 2014	8	Unknown/combined	First-onset schizophrenia (<5 y/drug naive)	Adults, 17–60	China (China)	55/50 (91)	18.1 (3.0)/NS	41.2 (13.3)	68/32	0/0/100	1.95 (1.6)/NR	NR
Duration: >13 wk												
Arango, 2009	24	Open-label/inpatient	First psychotic episode (<1 y/NS)	Adolescents, 12–18	Europe (Spain)	26/16 (62)	9.7 (6.5)/NS	15.7 (1.4)	77/23	77/0/23	NR/NR	21.7 (NR)
Findling, 2010	52	Double-blind/unknown	Early-onset schizophrenia spectrum disorder (NS/NS)	Children/adolescents, 8–19	USA (USA)	13/3 (23)	9.6 (5.4)/2.5-20	NR	92/8	69/23/8	NR/NR	21.7 (3.9)
Green, 2006/Zipursky, 2005	104	Double-blind/mixed	First-episode psychosis (<5 y/<16 wk)	Adolescents/adults, 16–40	International^b^	131/31 (23)	10.2 (NR)/5-20	23.5 (4.6)	79/21	51/37/11	1.0 (1.0)/1.3 (1.7)	23.6 (4.8)
Kahn, 2008	52	Open-label/mixed	First-episode schizophrenia and schizophreniform disorder (<2 y/<6 wk)	Adults, 18–40	Europe^d^	105/82 (78)	12.6 (4.7)/5-20	26.3 (5.9)	64/36	95/NR/5	NR/NR	22.0 (3.0)
McEvoy, 2007/Patel, 2009	52	Double-blind/mixed	Early psychosis (<5 y/<16 wk)	Adolescents/adults, 16–40	USA (USA)	133/42 (32)	11.7 (5.3)/2.5-20	24.7 (5.8)	76/24	46/46/8	0.92 (1.1)/1.73 (2.2)	25.8 (6.2)
Perez-Iglesias, 2008/Perez-Iglesias, 2009	52	Open-label/mixed	First-episode psychosis (1st episode/ drug naive)	Adolescents/adults, 15–60	Europe (Spain)	54/36 (67)	10.1 (3.9)/5-20	27.6 (6.9)	59/41	NR	1.0 (2.45)/NR	22.8 (2.9)
San, 2012	52	Open-label/combined	First-episode psychosis (NR/drug naive)	Adults, ≥18	Europe (Spain)	25/15 (60)	7.5 (6.3)/7.5-40	25.3 (6.8)	68/32	NR	1.34 (3.7)/NR	21.8 (2.9)

Abbreviations: AMI, amisulpride; AP, antipsychotic; BMI, body mass index; max, maximum; NR, not reported; NS, not stated; OLZ, olanzapine.

^*^Values reported as medians.

^a^Austria, Belgium, Bulgaria, Czech Republic, Denmark, France, Germany, Israel, Italy, Netherlands, Poland, Romania, Spain, Switzerland, UK.

^b^North America and Western Europe.

^c^North America and Europe.

^d^Austria, Belgium, Bulgaria, Czech Republic, France, Germany, Israel, Italy, Netherlands, Poland, Romania, Spain, Sweden, and Switzerland.

^e^Age group not reported but determined based on sample characteristics.

### Risk of Bias Assessment for Included Studies Assessing Olanzapine-Associated Weight Gain

The overall risk of bias was low for most RCTs assessing olanza­pine-associated weight gain that were included in the meta-analysis. Two RCTs were rated as having some concerns about bias (Findling et al., 2010; Kahn et al., 2018) and were excluded from the sensitivity analysis. The results of the risk-of-bias analysis are summarized in supplementary Table 2.

### Meta-Analysis of Weight Gain

**Figure 2.**
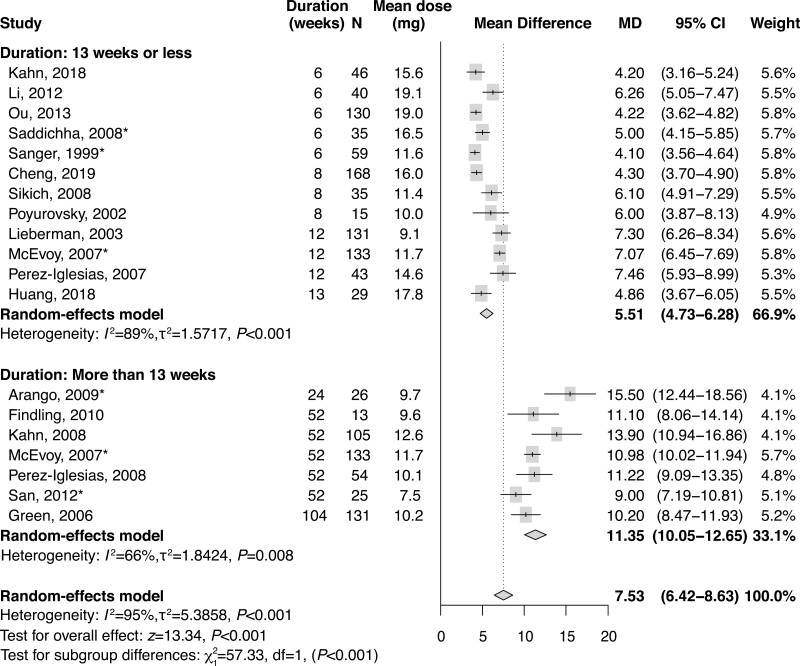
Forest plot of mean weight gain, grouped by study duration. *SDs were imputed using Bracken’s approach to estimate a coefficient of variation from available standard deviations, taking the mean difference into account. MD, mean difference.

Weight change data were available for 19 RCTs that randomized 1351 patients to olanzapine. Figure 2 presents a forest plot of overall mean weight gain as well as the mean weight gain observed separately in studies of 13 weeks or less and those longer than 13 weeks. Table 2 displays associated *P* values and Higgins *I*^*2*^ heterogeneity values. Across the 19 studies analyzed, the mean (95% CI) weight gain was 7.53 (6.42–8.63) kg. In studies ≤13 weeks (n = 12), mean (95% CI) weight gain was 5.51 (4.73–6.28) kg, but in those >13 weeks (n = 7), mean (95% CI) weight gain was >2 times higher (11.35 [10.05–12.65] kg), with the difference in weight gain between study durations being statistically significant (*P < *.001).

**Table 2.** Meta-Analysis of Weight Gain and Metabolic Parameters

Outcome/subgroup	No. of studies	Effect size		Subgroup comparison	Heterogeneity
		MD (95% CI)	*P*	*P*	*I* ^2^
Weight gain, kg					
All studies	19	7.53 (6.42–8.63)	<.01		95
≤13 wk	12	5.51 (4.73–6.28)	<.01	<.001	89
>13 wk	7	11.35 (10.05–12.65)	<.01		66
BMI, kg/m^2^					
All studies	12	2.39 (1.95–2.83)	<.001		97
≤13 wk	9	2.01 (1.72–2.30)	<.001	<.001	90
>13 wk	3	3.69 (3.45–3.94)	<.001		0
Waist circumference, cm					
All studies	3	5.48 (3.15–7.80)	<.001		88
≤13 wk	3	5.48 (3.15–7.80)	<.001	NA	88
>13 wk	0				
Blood glucose, mg/dL					
All studies	14	4.78 (2.07–7.49)	<.001		94
≤13 wk	9	4.01 (0.69–7.33)	.02	.24	93
>13 wk	5	6.47 (4.10–8.85)	<.001		62
Insulin, µU/mL					
All studies	8	6.17 (2.81–9.52)	<.001		97
≤13 wk	5	8.24 (2.23–14.25)	.007	.07	98
>13 wk	3	2.54 (1.80–3.27)	<.001		0
HOMA–IR					
All studies	5	0.75 (0.15–1.34)	.01		82
≤13 wk	4	0.73 (0.07–1.39)	.03	.81	86
>13 wk	1	0.90 (−0.30 to 2.10)	.14		NA
Total cholesterol, mg/dL					
All studies	12	18.88 (14.35–23.42)	<.001		88
≤13 wk	8	18.22 (12.56–23.89)	<.001	.80	90
>13 wk	4	19.73 (9.53–29.94)	<.001		85
Triglycerides, mg/dL					
All studies	12	38.61 (29.08–48.13)	<.001		86
≤13 wk	8	39.57 (26.33–52.81)	<.001	.81	86
>13 wk	4	36.40 (14.87–57.93)	<.001		90
LDL cholesterol, mg/dL					
All studies	8	13.54 (8.23–18.84)	<.001		95
≤13 wk	5	10.85 (4.84–16.85)	<.001	.17	96
>13 wk	3	19.28 (8.94–29.63)	<.001		75
HDL cholesterol, mg/dL					
All studies	11	−2.18 (−4.76 to 0.41)	.10		97
≤13 wk	7	−0.76 (−4.33 to 2.82)	.68	.06	96
>13 wk	4	−4.61 (−6.52 to −2.71)	<.001		73

Abbreviations: BMI, body mass index; HDL, high-density lipoprotein; HOMA-IR, Homeostatic Model Assessment for Insulin Resistance; LDL, low-density lipoprotein; MD, mean difference; NA, not applicable.

For the subgroup analyses, there was an interaction for weight gain by region in ≤13-week studies (*P* = .02) but not in >13-week studies (*P* = .48). There were no differences in weight gain by maximum prior antipsychotic exposure (≤13 weeks, *P* = .72; >13 weeks, *P* = .16). For studies ≤13 weeks in duration (1 conducted in children and adolescents, 7 in a mixed adolescent/adult population, and 4 in adults only), there was a trend level interaction between weight gain and target age (*P* = .05), with numerically more weight gain in children and adolescents as well as in mixed adolescent/adult populations compared with adult populations. This interaction was not observed for studies longer than 13 weeks (*P* = .54), which included 2 studies in children and adolescents, 3 in a mixed adolescent/adult population, and 2 in adults (data not shown). An additional subgroup analysis revealed no differences in weight gain between patients who were antipsychotic naive vs those who had prior antipsychotic exposure (≤13-week studies, *P* = .81; >13-week studies, *P* = .16).

### Meta-Analysis of Metabolic Parameters

Olanzapine administration was associated with changes in most metabolic parameter values that occurred early in treatment (Table 2). The only difference by study duration was in BMI. The meta-analytic mean (95% CI) BMI increase was 2.39 (1.95–2.83) kg/m^2^ across all analyzed studies (n = 12). Consistent with results for weight gain, the mean (95% CI) increases in BMI were larger in >13-week studies than in those lasting ≤13 weeks: 3.69 (3.45–3.94) vs 2.01 (1.72–2.30) kg/m^2^, respectively (*P < *.001).

No studies >13 weeks evaluated waist circumference. In ≤13-week studies, the mean (95% CI) increase in waist circumference with olanzapine treatment was 5.48 (3.15–7.80) cm (*P < *.001). For other metabolic parameters, increases in total cholesterol, triglycerides, LDL cholesterol, blood glucose, insulin, and HOMA-IR were reported across all studies (except 1 study ≥13 weeks, where no difference in HOMA-IR was reported). HDL cholesterol was decreased in studies >13 weeks but not in those lasting ≤13 weeks.

### Meta-Regression of Weight Gain and Metabolic Parameters

Meta-regression results evaluating the influence of study and patient characteristics on olanzapine-associated weight gain and metabolic parameters are shown in Table 3. The RCT duration in weeks was not a feasible moderator variable for >13-week studies, as these studies were mostly 52 weeks long; therefore, there was too little variability to conduct the analysis. In ≤13-week studies, RCT duration was associated with weight gain (coefficient = 0.30, 95% CI = 0.11–0.50, *P* = .002). However, no associations were found in ≤13-week studies between weight gain and mean olanzapine dose, study completion rate, mean age of patients, or percentage of male patients enrolled. The mean age of enrolled patients was associated with changes in BMI (coefficient = −0.10, 95% CI = −0.17 to −0.03, *P < *.01), triglycerides (coefficient = 1.74, 95% CI = 0.29–3.20, *P* = .02), and LDL cholesterol (coefficient = −0.52, 95% CI = −0.65 to −0.38, *P < *.001).

**Table 3.** Meta-Regression of Weight Gain and Metabolic Parameters

Outcome	Duration ≤13 wk			Duration >13 wk^a^		
	No. of studies	Coefficient (95% CI)	*P* value	No. of studies	Coefficient (95% CI)	*P* value
Weight gain, kg						
Duration, wk	12	0.30 (0.11 to 0.50)	.002	NA	–	–
Mean dose, mg	12	−0.17 (−0.40 to 0.05)	.13	7	0.59 (−0.30 to 1.48)	.19
Completion rate, %	10	−0.002 (−0.060 to 0.055)	.93	7	0.03 (−0.02 to 0.09)	.25
Mean age, y	10	−0.16 (−0.59 to 0.28)	.47	6	−0.35 (−0.75 to 0.05)	.08
Male, %	11	0.06 (−0.02 to 0.14)	.15	7	−0.01 (−0.15 to 0.14)	.93
BMI, kg/m^2^						
Duration, wk	9	0.06 (−0.10 to 0.22)	.48	NA	–	–
Mean dose, mg	9	−0.04 (−0.18 to 0.10)	.58	3	0.11 (−0.50 to 0.73)	.71
Completion rate, %	8	−0.01 (−0.03 to 0.01)	.44	3	0.01 (−0.01 to 0.03)	.33
Mean age, y	8	−0.10 (−0.17 to −0.03)	.008	0	–	–
Male, %	9	0.031 (−0.01 to 0.07)	.12	3	−0.02 (−0.06 to 0.01)	.19
Blood glucose, mg/dL						
Duration, wk	9	−0.39 (−1.58 to 0.81)	.52	NA	–	–
Mean dose, mg	9	0.72 (−0.02 to 1.46)	.057	5	1.01 (−0.01 to 2.04)	.053
Completion rate, %	7	0.00 (−0.23 to 0.23)	1	5	0.01 (−0.13 to 0.14)	.94
Mean age, y	7	0.17 (−0.45 to 0.79)	.59	4	−1.44 (−3.79 to 0.91)	.23
Male, %	8	−0.01 (−0.43 to 0.41)	.97	5	−0.01 (−0.25 to 0.24)	.95
Insulin (µU/mL)						
Duration, wk	5	−1.20 (−3.51 to 1.11)	.31	NA	–	–
Mean dose, mg	5	−1.25 (−3.87 to 1.37)	.35	3	−0.14 (−1.08 to 0.80)	.77
Completion rate, %	5	−0.13 (−0.56 to 0.30)	.55	3	−0.01 (−0.06 to 0.05)	.87
Mean age, y	4	1.03 (−0.18 to 2.24)	.10	0	–	–
Male, %	5	0.20 (−1.02–1.42)	.74	3	0.004 (−0.11 to 0.12)	.95
HOMA-IR						
Duration, wk	4	−0.05 (−0.25 to 0.15)	.64	NA	–	–
Mean dose, mg	4	−0.01 (−0.23 to 0.20)	.90	1	–	–
Completion rate, %	4	−0.02 (−0.04 to 0.003)	.08	1	–	–
Mean age, y	3	−0.19 (−0.47 to 0.10)	.21	1	–	–
Male, %	4	0.05 (−0.03 to 0.13)	.19	1	–	–
Total cholesterol, mg/dL						
Duration, wk	8	0.12 (−2.56 to 2.79)	.93	NA	–	–
Mean dose, mg	8	−0.21 (−2.04 to 1.62)	.82	4	5.75 (−3.17 to 14.67)	.21
Completion rate, %	7	0.03 (−0.39 to 0.46)	.88	4	0.34 (0.17 to 0.51)	<.001
Mean age, y	7	−0.49 (−1.19 to 0.22)	.18	3	3.27 (−5.21 to 11.74)	.45
Male, %	8	−0.22 (−1.03 to 0.60)	.60	4	−0.84 (−1.46 to −0.22)	.008
Triglycerides, mg/dL						
Duration, wk	8	−1.63 (−5.90 to 2.65)	.46	NA	–	–
Mean dose, mg	8	2.87 (−1.38 to 7.13)	.19	4	6.00 (−18.82 to 30.82)	.64
Completion rate, %	6	0.34 (−0.41 to 1.10)	.38	4	−0.426 (−1.17 to 0.32)	.26
Mean age, y	6	1.74 (0.29 to 3.20)	.019	3	−13.56 (−29.34 to 2.21)	.09
Male, %	7	−0.26 (−1.82–1.30)	.74	4	0.16 (−1.60 to 1.92)	.86
LDL cholesterol, mg/dL						
Duration, wk	5	0.83 (−1.37 to 3.03)	.46	NA	–	–
Mean dose, mg	5	−1.02 (−2.92 to 0.87)	.29	3	4.89 (1.45 to 8.33)	.005
Completion rate, %	5	−0.13 (−0.42 to 0.16)	.39	3	0.35 (−0.08 to 0.77)	.11
Mean age, y	4	−0.52 (−0.65 to −0.38)	<.001	0	–	–
Male, %	5	0.29 (−0.77 to 1.34)	.59	3	−0.35 (−1.28 to 0.57)	.45
HDL cholesterol, mg/dL						
Duration, wk	7	−0.07 (−1.05 to 0.91)	.89	NA	–	–
Mean dose, mg	7	0.39 (−0.37 to 1.15)	.31	4	−0.36 (−2.18 to 1.46)	.70
Completion rate, %	5	−0.05 (−0.32 to 0.22)	.72	4	0.04 (−0.03 to 0.11)	.22
Mean age, y	5	−0.71 (−1.56 to 0.150)	.11	3	1.51 (0.69 to 2.34)	<.001
Male, %	6	−0.11 (−0.57 to 0.35)	.63	4	−0.05 (−0.22 to 0.11)	.51

Abbreviations: BMI, body mass index; HDL, high-density lipoprotein; HOMA-IR, Homeostatic Model Assessment for Insulin Resistance; LDL, low-density lipoprotein; NA, not applicable.

^a^All studies with duration >13 weeks reporting on secondary outcomes have similar duration (52 weeks); thus, duration in weeks is not a feasible moderator variable.

In >13-week studies, total cholesterol concentration was associated with completion rate (coefficient = 0.34, 95% CI = 0.17–0.51, *P < *.001) and with the percentage of male patients enrolled (coefficient = −0.84, 95% CI = −1.46 to −0.22, *P* = .008). LDL cholesterol concentration was associated with mean olanzapine dose (coefficient = 4.89, 95% CI = 1.45–8.33, *P* = .005). HDL cholesterol concentration was associated with the mean age of patients (coefficient = 1.51, 95% CI = 0.69–2.34, *P < *.001). Changes in BMI, blood glucose, insulin levels, and triglycerides were not associated with mean olanzapine dose, completion rates, mean age of patients, or percentage of male patients enrolled. HOMA-IR was not assessed in meta-regression, as only 1 study >13 weeks long reported on this outcome.

Additional analyses explored the relationship between weight gain and metabolic changes. Across all trials, no correlations were observed between weight gain and changes in metabolic parameters, except HDL cholesterol (coefficient = −0.95, *R*^*2*^ = 0.60, *P* = .01). However, this correlation was not observed when studies ≤13 weeks and those >13 weeks long were analyzed separately (Table 4).

**Table 4.** Correlation of Mean Weight Gain With Mean Change in Metabolic Parameters

Parameter	All trials				Duration ≤13 wk				Duration >13 wk			
	No. of studies	Coefficient	*P*	R^2^	No. of studies	Coefficient	*P*	R^2^	No. of studies	Coefficient	*P*	R^2^
Blood glucose	12	0.27	.39	0.08	7	−1.60	.06	0.54	5	0.73	.43	0.22
Insulin	7	−0.55	.37	0.16	4	−0.60	.87	0.02	3	−0.19	.56	0.41
HOMA-IR	5	−0.03	.80	0.03	4	−0.21	.40	0.36	1	NA	NA	NA
Total cholesterol	10	0.21	.84	0.01	6	−0.9	.64	0.06	4	6.58	.32	0.46
Triglycerides	10	−0.78	.65	0.03	6	−4.51	.39	0.19	4	−4.54	.71	0.08
LDL cholesterol	7	0.98	.16	0.35	4	1.56	.12	0.78	3	5.57	.23	0.88
HDL cholesterol	10	−0.95	.01	0.60	6	−2.03	.11	0.52	4	0.29	.75	0.06

Abbreviations: HDL, high-density lipoprotein; HOMA-IR, Homeostatic Model Assessment for Insulin Resistance; LDL, low-density lipoprotein; NA, not applicable.

### Sensitivity Analysis and Funnel Plot Assessment

Results from the sensitivity analysis were similar to those of the primary analysis of olanzapine-associated weight gain (supplementary Figure 1). Among RCTs with a low risk of bias (n = 16), the mean (95% CI) weight gain was 7.57 (6.40–8.74) kg in patients randomized to olanzapine. Supplementary Table 3 displays the results of Egger’s test of funnel plot asymmetry. The parameters of weight, BMI, blood glucose, total cholesterol, triglycerides, and HDL cholesterol were included as each had sufficient studies (≥10) to test for potential publication bias and study heterogeneity. Egger’s test was significant for changes in weight and BMI; funnel plots (supplementary Figures 2 and 3, respectively) confirmed asymmetry for these parameters but did not reveal indications of publication bias, as there were few studies with large standard errors. Thus, funnel plot asymmetry is likely driven by heterogeneity of the included RCTs and their patient characteristics.

## DISCUSSION

The results of this meta-analysis indicate that olanzapine is associated with clinically meaningful and statistically significant weight gain in patients with first-episode psychosis or early-phase schizophrenia and that increases in weight and in BMI are higher in studies lasting longer than 13 weeks. In this analysis, olanza­pine treatment is also associated with changes to glycemic and lipid indices, regardless of study duration. While this analysis confirms the findings of rapidly evolving weight gain and metabolic changes after the initiation of olanzapine treatment, as reported previously (Citrome et al., 2011; Correll et al., 2020), there were no clear relationships between weight gain and metabolic sequelae in this sample of clinical studies of limited duration.

The mean difference for olanzapine-associated weight gain was 7.53 kg across all RCTs included in the analysis. Stratified by duration, weight gain was more than twice as high in studies lasting >13 weeks than in those lasting ≤13 weeks (11.35 vs 5.51 kg). Overall, the mean difference in BMI associated with olanza­pine treatment was 2.39 kg/m^2^. Like weight gain, this increase was higher in studies >13 weeks (3.69 kg/m^2^) compared with those lasting ≤13 weeks (2.01 kg/m^2^). Although no studies >13 weeks analyzed changes in waist circumference, the mean increase was 5.48 cm for studies ≤13 weeks. In addition, there were generally small increases from baseline in most glycemic and lipid parameters in studies ≤13 weeks, but these increases did not appear to worsen with longer treatment exposures in studies lasting >13 weeks.

The observed weight-related outcomes are clinically significant because weight gain and elevated BMI are known drivers of cardiometabolic risk, including diabetes mellitus, dyslipidemia, and metabolic syndrome (Newcomer, 2005; De Hert et al., 2011). Epidemiologic studies suggest that the addition of 5 kg of body weight or a 1-kg/m^2^ increase in BMI is sufficient to raise the risk of developing adverse cardiovascular conditions (Zheng et al., 2017; Choi et al., 2018; Larsson et al., 2020). Likewise, an increase of ≥5 cm in waist circumference is associated with a higher risk of mortality, independent of BMI (Cerhan et al., 2014). Based on the meta-analytic evidence, these thresholds were reached in studies ≤13 weeks and were surpassed in studies lasting >13 weeks, suggesting that patients with first-episode psychosis or early-phase schizophrenia may be vulnerable to olanzapine-associated weight gain in both the early and later stages of treatment.

Because of the association between weight gain and cardiometabolic risk, the weight and metabolic effects of olanzapine treatment are often assumed to be related because they occur in tandem. However, acute cardiometabolic changes caused by antipsychotic medications may occur through direct molecular effects, while delayed changes may occur via indirect effects associated with weight gain and increases in central adiposity (Maayan et al., 2010; De Hert et al., 2012). The meta-analytic evidence presented herein suggests that, in the context of clinical trials in patients with first-episode psychosis or early-phase schizophrenia, evaluation of the metabolic risk of olanzapine treatment likely requires studies of longer duration. Changes in HOMA-IR, total cholesterol, triglycerides, HDL cholesterol, and LDL cholesterol do not differ between studies lasting ≤13 and >13 weeks despite the observation that studies lasting >13 weeks are associated with greater increases in weight and BMI. Although the clinical relevance of these metabolic changes in an early-in-illness population is unclear, these data are consistent with previous studies in healthy volunteers (Albaugh et al., 2011; Toledo et al., 2022) and patients with schizophrenia (Chiu et al., 2010; Correll et al., 2020), in which differences from baseline in markers of glucose and lipid metabolism were observed within weeks or even days of olanzapine initiation.

In addition, no correlations were observed between weight gain and metabolic parameter changes in studies ≤13 or >13 weeks; a correlation was observed for HDL cholesterol when analyzed across all studies but not when ≤13- and >13-week studies were evaluated separately. There is abundant epidemiologic evidence supporting the hypothesis that persistent weight gain leads to cardiometabolic sequelae (De Hert et al., 2011). Indeed, differences from baseline in glucose and lipid metabolism observed in the current analysis are somewhat sustained and may mask more subtle, weight-dependent effects. Secondary weight-related metabolic disturbances may emerge gradually over time, especially in these early-in-illness patients who only recently started receiving olanzapine treatment. In this regard, olanzapine may elicit initial, weight-independent metabolic changes that give way to more pathologic, weight-dependent changes over time. However, the studies lasting >13 weeks included in this analysis may not have been of adequate duration to detect metabolic sequelae in this specific patient population, as only 1 RCT included here went on for longer than 1 year.

Increases in weight and BMI after the initiation of olanza­pine treatment limit its clinical utility and may lead to untoward cardiometabolic consequences. In the included RCTs, there was minimal evidence of a relationship between weight gain and symptom improvement, suggesting that olanzapine’s clinical efficacy may be independent of its effects on weight in this early-in-illness population. Given olanzapine’s similar efficacy to other atypical antipsychotics in the treatment of positive symptoms in first-episode schizophrenia (Zhu et al., 2017) and its potential causation of cardiometabolic sequalae, olanzapine is not recommended as a first-line treatment option for early-episode schizophrenia (Buchanan et al., 2010).

Weight gain experiences among patients who are early in their course of illness and initiate treatment with olanzapine may impact treatment adherence. Efforts to address weight and/or BMI increases by switching to a different antipsychotic agent may introduce additional untoward side effects or inadequate symptom control, resulting in treatment discontinuation and potential for disease relapse, hospitalization, and disease worsening. From the perspective of patients’ longer-term health, it is important to understand the timing of metabolic changes and their relationship to weight gain; such understanding may help to inform treatment choices for patients who are early in their illness. Being able to provide treatments that limit weight gain and the potential for medication-associated cardiometabolic sequelae for first-episode psychosis or early-in-illness patients is an important consideration for those requiring long-term continuity of pharmacologic and psychosocial treatment (Firth et al., 2019).

### Limitations

Several study limitations should be noted. Although weight gain associated with olanzapine is a well-known effect, we identified relatively few RCTs that reported on the cardiometabolic outcomes (including waist circumference) associated with olanza­pine treatment. For example, when stratified by duration, fewer than 10 studies were identified that reported on cardiometabolic parameters of interest for this meta-analysis and meta-regression. As mentioned above, there was significant between-study heterogeneity in our analyses. Between-study variability can be expected because of the varying patient demographics (children, adolescents, adults) and regions included in the meta-analysis. In addition, different definitions of first-episode psychosis or early-phase schizophrenia were used across individual studies. Whereas all studies enrolled patients who were early in their illness, some studies defined the inclusion period from onset of symptoms or diagnosis, while other studies defined it by the time since the first antipsychotic treatment or lack thereof. Most of the included RCTs did not report on the duration of untreated psychosis, precluding any meaningful analysis of its effect on olanzapine-associated weight gain. Thus, it is unclear what impact, if any, the timing of treatment initiation had on weight and cardiometabolic outcomes.

Also potentially contributing to between-study variability were differences in collecting and reporting on cardiometabolic endpoints. For example, although patients were requested to fast before blood samples for metabolic parameters were obtained, fasting status was not confirmed. This variability in study and patient characteristics was evident as asymmetry in the funnel plots of weight gain and of BMI. Furthermore, subgroup analyses of weight gain by trial duration, region, and prior antipsychotic exposure were few, thus limiting their statistical power to show differences. Therefore, the results of subgroup analyses and the influence of study and patient characteristics on olanzapine-associated cardiometabolic outcomes should be interpreted with caution. Lastly, few studies included non-White patients; as such, these results may not generalize to those of non-White races. Despite these limitations, this meta-analysis provides evidence of consistent weight gain, as well as early changes in cardiometabolic indices, due to olanzapine treatment in patients with first-episode psychosis or early-phase schizophrenia.

## CONCLUSIONS

Across clinical studies conducted in patients with first-episode psychosis or early-phase schizophrenia, olanzapine was associated with weight gain that increased with trial duration. There were changes from baseline in several different cardiometabolic parameters that were observed across all study durations. The lack of correlations between weight gain and worsening of metabolic parameters may have been impacted by the population studied (ie, patients with early-phase illness), the fact that only 1 of the studies was longer than 12 months, and that most (12 of 19) included studies assessing olanzapine-associated weight gain were ≤13 weeks long. This meta-analysis confirms that patients with early-episode psychosis are vulnerable to olanzapine-associated weight gain and metabolic sequelae. Because long-term antipsychotic treatment is recommended in schizophrenia treatment guidelines, strategies that minimize weight gain should be carefully considered in the context of not only first-episode psychosis but also chronic illness to reduce the propensity for metabolic sequelae to evolve into cardiometabolic morbidities.

## Supplementary Material

pyad029_suppl_Supplementary_Material

## Registration and Protocol

This systematic review and meta-analysis were not registered. All elements of the protocol are included in the manuscript.

## Interest Statement

C.U.C. has been a consultant and/or advisor to or has received honoraria from AbbVie, Acadia, Alkermes, Allergan, Angelini, Aristo, Axsome, Boehringer-Ingelheim, Cardio Diagnostics, Cerevel, CNX Therapeutics, Compass Pathways, Damitsa, Gedeon Richter, Hikma, Holmusk, Intra-Cellular Therapies, Janssen/J&J, Karuna, LB Pharma, Lundbeck, MedAvante-ProPhase, MedinCell, Medscape, Merck, MindPax, Mitsubishi Tanabe Pharma, Mylan, Neurocrine, Noven, Otsuka, Pfizer, Pharmabrain, PPD Biotech, Recordati, Relmada, Reviva, Rovi, Seqirus, Servier, SK Life Science, Sumitomo Dainippon, Sunovion, Sun Pharma, Supernus, Takeda, Teva, and Viatris. He provided expert testimony for Janssen and Otsuka. He served on a Data Safety Monitoring Board for Lundbeck, Relmada, Reviva, Rovi, and Teva. He has received grant support from Janssen and Takeda. He received royalties from UpToDate and is also a stock option holder of Cardio Diagnostics, MindPax, and LB Pharma. M.H. has received honoraria for consultancy/speaking from the Lundbeck Foundation and Otsuka, unrelated to this project. C.G., M.S.T., D.M., and A.S. are employees of Alkermes, Inc., and may own stock/options in the company.
