# Supplementary material for: Weight Gain and Metabolic Changes in Patients With First-Episode Psychosis or Early-Phase Schizophrenia Treated With Olanzapine: A Meta-Analysis
Source: Int J Neuropsychopharmacol. 2023 Jun 16;26(7):451–64. doi: 10.1093/ijnp/pyad029 (PMC10388390; doi:10.1093/ijnp/pyad029)
Supplement: pyad029_suppl_Supplementary_Material [file pyad029_suppl_supplementary_material.docx]

# SUPPLEMENTAL MATERIAL

## Supplementary Table S1. PRISMA checklist

| **Section and Topic** | **Item #** | **Checklist item** | **Location where item**  **is reported** |
| --- | --- | --- | --- |
| **TITLE** | | |  |
| Title | 1 | Identify the report as a systematic review. | 1 |
| **ABSTRACT** | | |  |
| Abstract | 2 | See the PRISMA 2020 for Abstracts checklist. | 2-3 |
| **INTRODUCTION** | | |  |
| Rationale | 3 | Describe the rationale for the review in the context of existing knowledge. | 4-5 |
| Objectives | 4 | Provide an explicit statement of the objective(s) or question(s) the review addresses. | 5 |
| **METHODS** | | |  |
| Eligibility criteria | 5 | Specify the inclusion and exclusion criteria for the review and how studies were grouped for the syntheses. | 6-7 |
| Information sources | 6 | Specify all databases, registers, websites, organisations, reference lists and other sources searched or consulted to identify studies. Specify the date when each source was last searched or consulted. | 6 |
| Search strategy | 7 | Present the full search strategies for all databases, registers and websites, including any filters and limits used. | 6 |
| Selection process | 8 | Specify the methods used to decide whether a study met the inclusion criteria of the review, including how many reviewers screened each record and each report retrieved, whether they worked independently, and if applicable, details of automation tools used in the process. | 7 |
| Data collection process | 9 | Specify the methods used to collect data from reports, including how many reviewers collected data from each report, whether they worked independently, any processes for obtaining or confirming data from study investigators, and if applicable, details of automation tools used in the process. | 7 |
| Data items | 10a | List and define all outcomes for which data were sought. Specify whether all results that were compatible with each outcome domain in each study were sought (e.g. for all measures, time points, analyses), and if not, the methods used to decide which results to collect. | 8 |
|  | 10b | List and define all other variables for which data were sought (e.g. participant and intervention characteristics, funding sources). Describe any assumptions made about any missing or unclear information. | 7-8 |
| Study risk of bias assessment | 11 | Specify the methods used to assess risk of bias in the included studies, including details of the tool(s) used, how many reviewers assessed each study and whether they worked independently, and if applicable, details of automation tools used in the process. | 8 |
| Effect measures | 12 | Specify for each outcome the effect measure(s) (e.g. risk ratio, mean difference) used in the synthesis or presentation of results. | 8-10 |
| Synthesis methods | 13a | Describe the processes used to decide which studies were eligible for each synthesis (e.g. tabulating the study intervention characteristics and comparing against the planned groups for each synthesis (item #5)). | 8-10 |
|  | 13b | Describe any methods required to prepare the data for presentation or synthesis, such as handling of missing summary statistics, or data conversions. | 8-10 |
|  | 13c | Describe any methods used to tabulate or visually display results of individual studies and syntheses. | 8-10 |
|  | 13d | Describe any methods used to synthesize results and provide a rationale for the choice(s). If meta-analysis was performed, describe the model(s), method(s) to identify the presence and extent of statistical heterogeneity, and software package(s) used. | 8-10 |
|  | 13e | Describe any methods used to explore possible causes of heterogeneity among study results (e.g. subgroup analysis, meta-regression). | 8-10 |
|  | 13f | Describe any sensitivity analyses conducted to assess robustness of the synthesized results. | 9 |
| Reporting bias assessment | 14 | Describe any methods used to assess risk of bias due to missing results in a synthesis (arising from reporting biases). | 8, 11 |
| Certainty assessment | 15 | Describe any methods used to assess certainty (or confidence) in the body of evidence for an outcome. | 8-10 |

NA, not applicable.

## Supplementary Table S2 . Risk of Bias Assessment for Included Studies Assessing Olanzapine-Associated Weight Gain

| Author/year^a^ | Bias arising from the randomization process | Bias due to deviations from intended interventions | Bias due to missing outcome data | Bias in measurement of the outcome | Bias in selection of the reported result | Overall risk of bias |
| --- | --- | --- | --- | --- | --- | --- |
| (Arango et al., 2009) | Low | Some concerns | Low | Low | Low | Low |
| (Cheng et al., 2019) | Low | Some concerns | Low | Low | Low | Low |
| (Findling et al., 2010) | Low | Low | Some concerns | Low | Low | Some concerns |
| (Green et al., 2006) | Low | Low | Low | Low | Low | Low |
| (Huang et al., 2018) | Low | Some concerns | Low | Low | Low | Low |
| (Kahn et al., 2008) | Low | Some concerns | Low | Low | Low | Low |
| (Kahn et al., 2018) | Low | Some concerns | Low | Low | Low | Some concerns |
| (Li et al., 2012) | Low | Some concerns | Low | Low | Low | Low |
| (Lieberman et al., 2003) | Low | Low | Low | Low | Low | Low |
| (McEvoy et al., 2007)^b^ | Low | Low | Some concerns | Low | Low | Low |
| (Ou et al., 2013) | Low | Some concerns | Low | Low | Low | Low |
| (Perez-Iglesias et al., 2007) | Low | Some concerns | Low | Low | Low | Low |
| (Perez-Iglesias et al., 2008) | Low | Some concerns | Low | Low | Low | Low |
| (Poyurovsky et al., 2002) | Low | Low | Low | Some Concerns | Low | Low |
| (Saddichha et al., 2008b) | Low | Low | Low | Low | Low | Low |
| (San et al., 2012) | Low | Some concerns | Low | Low | Low | Low |
| (Sanger et al., 1999) | Some concerns | Low | Low | Low | Low | Low |
| (Sikich et al., 2008) | Low | Low | Some concerns | Low | Low | Low |

^a^The analysis was conducted for 18 primary studies assessing olanzapine-associated weight gain, and not for related studies derived from the primary study.

^b^Provided ≤13 and >13 weeks of data; thus, 19 studies informed the olanzapine-associated weight gain analysis.

## Supplementary Table S3. Egger’s Test of Funnel Plot Asymmetry

| **Outcome** | **No. of studies** | **Egger’s test *P* value** | **No. of studies plus imputed** | **Main analysis MD (95% CI)** | **Trim-and-fill MD (95% CI)** |
| --- | --- | --- | --- | --- | --- |
| Weight gain, kg | 19 | 0.002 | 27 | 7.53 (6.42-8.63) | 5.21 (4.02-6.41) |
| BMI, kg/m^2^ | 12 | 0.04 | 17 | 2.39 (1.95-2.83) | 1.80 (1.34-2.25) |
| Waist circumference, cm | 3 | NA | NA | NA | NA |
| Blood glucose, mg/dL | 14 | 0.052 | 21 | 4.78 (2.07-7.49) | 0.78 (−2.01-3.58) |
| Insulin, µU/mL | 8 | NA | NA | NA | NA |
| HOMA-IR | 5 | NA | NA | NA | NA |
| Total cholesterol, mg/dL | 12 | 0.34 | 15 | 18.88 (14.35-23.42) | 22.38 (17.47-27.29) |
| Triglycerides, mg/dL | 12 | 0.07 | 17 | 38.61 (29.08-48.13) | 25.80 (15.41-36.19) |
| LDL cholesterol | 8 | NA | NA | NA | NA |
| HDL cholesterol | 11 | 0.43 | 12 | –2.18 (−4.76-0.41) | −1.26 (−3.98-1.46) |

BMI, body mass index; HDL, high-density lipoprotein; HOMA-IR, Homeostatic Model Assessment for Insulin Resistance; LDL, low-density lipoprotein; MD, mean difference; NA, not applicable.

## Supplementary Figure 1. Sensitivity Analysis for Weight Gain, Including Only Studies With Low Risk of Bias

MD, mean difference

## Supplementary Figure 2. Funnel Plot for Weight Gain


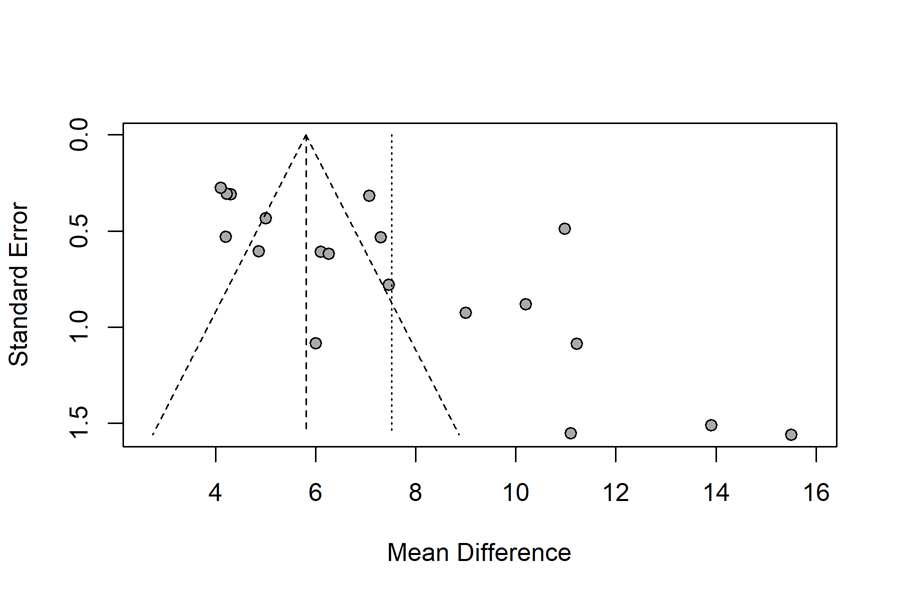


## Supplementary Figure 3. Funnel Plot for Body Mass Index


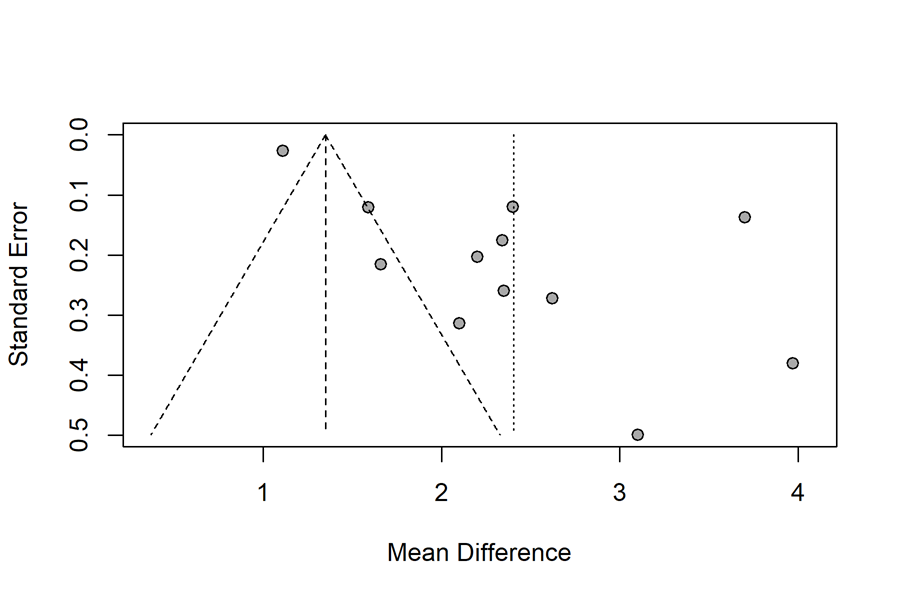


# REFERENCES

Albaugh VL, Singareddy R, Mauger D, Lynch CJ (2011) A double blind, placebo-controlled, randomized crossover study of the acute metabolic effects of olanzapine in healthy volunteers. PLoS One 6:e22662.

Arango C, Robles O, Parellada M, Fraguas D, Ruiz-Sancho A, Medina O, Zabala A, Bombín I, Moreno D (2009) Olanzapine compared to quetiapine in adolescents with a first psychotic episode. Eur Child Adolesc Psychiatry 18:418-428.

Bessonova L, Velligan DI, Weiden PJ, O'Sullivan AK, Yarlas A, Bayliss M, Baranwal N, Rychlec K, Carpenter-Conlin J, Doane MJ, Sajatovic M (2020) Antipsychotic treatment experiences of people with bipolar I disorder: patient perspectives from an online survey. BMC Psychiatry 20:354.

Bracken MB. Statistical methods for analysis of effects of treatment in overviews of randomized trials. In: Sinclair JC, Bracken MB, eds. Effective Care of the Newborn Infant Oxford: Oxford University Press; 1992:13-20.

Buchanan RW, Kreyenbuhl J, Kelly DL, Noel JM, Boggs DL, Fischer BA, Himelhoch S, Fang B, Peterson E, Aquino PR, Keller W (2010) The 2009 schizophrenia PORT psychopharmacological treatment recommendations and summary statements. Schizophr Bull 36:71-93.

Cerhan JR et al. (2014) A pooled analysis of waist circumference and mortality in 650,000 adults. Mayo Clin Proc 89:335-345.

Cheng Z, Yuan Y, Han X, Yang L, Cai S, Yang F, Lu Z, Wang C, Deng H, Zhao J, Xiang Y, Correll CU, Yu X (2019) An open-label randomised comparison of aripiprazole, olanzapine and risperidone for the acute treatment of first-episode schizophrenia: eight-week outcomes. J Psychopharmacol 33:1227-1236.

Chiu CC, Chen CH, Chen BY, Yu SH, Lu ML (2010) The time-dependent change of insulin secretion in schizophrenic patients treated with olanzapine. Prog Neuropsychopharmacol Biol Psychiatry 34:866-870.

Choi S, Kim K, Kim SM, Lee G, Jeong SM, Park SY, Kim YY, Son JS, Yun JM, Park SM (2018) Association of obesity or weight change with coronary heart disease among young adults in South Korea. JAMA Intern Med 178:1060-1068.

Cipriani A, Barbui C, Salanti G, Rendell J, Brown R, Stockton S, Purgato M, Spineli LM, Goodwin GM, Geddes JR (2011) Comparative efficacy and acceptability of antimanic drugs in acute mania: a multiple-treatments meta-analysis. Lancet 378:1306-1315.

Citrome L, Holt RI, Walker DJ, Hoffmann VP (2011) Weight gain and changes in metabolic variables following olanzapine treatment in schizophrenia and bipolar disorder. Clin Drug Investig 31:455-482.

Correll CU, Manu P, Olshanskiy V, Napolitano B, Kane JM, Malhotra AK (2009) Cardiometabolic risk of second-generation antipsychotic medications during first-time use in children and adolescents. JAMA 302:1765-1773.

Correll CU, Newcomer JW, Silverman B, DiPetrillo L, Graham C, Jiang Y, Du Y, Simmons A, Hopkinson C, McDonnell D, Kahn RS (2020) Effects of olanzapine combined with samidorphan on weight gain in schizophrenia: a 24-week phase 3 study. Am J Psychiatry 177:1168-1178.

Correll CU, Robinson DG, Schooler NR, Brunette MF, Mueser KT, Rosenheck RA, Marcy P, Addington J, Estroff SE, Robinson J, Penn DL, Azrin S, Goldstein A, Severe J, Heinssen R, Kane JM (2014) Cardiometabolic risk in patients with first-episode schizophrenia spectrum disorders: baseline results from the RAISE-ETP study. JAMA Psychiatry 71:1350-1363.

Correll CU, Solmi M, Veronese N, Bortolato B, Rosson S, Santonastaso P, Thapa-Chhetri N, Fornaro M, Gallicchio D, Collantoni E, Pigato G, Favaro A, Monaco F, Kohler C, Vancampfort D, Ward PB, Gaughran F, Carvalho AF, Stubbs B (2017) Prevalence, incidence and mortality from cardiovascular disease in patients with pooled and specific severe mental illness: a large-scale meta-analysis of 3,211,768 patients and 113,383,368 controls. World Psychiatry 16:163-180.

De Hert M, Correll CU, Bobes J, Cetkovich-Bakmas M, Cohen D, Asai I, Detraux J, Gautam S, Moller HJ, Ndetei DM, Newcomer JW, Uwakwe R, Leucht S (2011) Physical illness in patients with severe mental disorders. I. Prevalence, impact of medications and disparities in health care. World Psychiatry 10:52-77.

De Hert M, Detraux J, van Winkel R, Yu W, Correll CU (2012) Metabolic and cardiovascular adverse effects associated with antipsychotic drugs. Nat Rev Endocrinol 8:114-126.

DerSimonian R, Laird N (1986) Meta-analysis in clinical trials. Control Clin Trials 7:177-188.

Doane MJ, Sajatovic M, Weiden PJ, O'Sullivan AK, Maher S, Bjorner JB, Sikora Kessler A, Carpenter-Conlin J, Bessonova L, Velligan DI (2020) Antipsychotic treatment experiences of people with schizophrenia: patient perspectives from an online survey. Patient Prefer Adherence 14:2043-2054.

Duval S, Tweedie R (2000) Trim and fill: A simple funnel-plot-based method of testing and adjusting for publication bias in meta-analysis. Biometrics 56:455-463.

Egger M, Davey Smith G, Schneider M, Minder C (1997) Bias in meta-analysis detected by a simple, graphical test. BMJ 315:629-634.

Findling RL, Johnson JL, McClellan J, Frazier JA, Vitiello B, Hamer RM, Lieberman JA, Ritz L, McNamara NK, Lingler J, Hlastala S, Pierson L, Puglia M, Maloney AE, Kaufman EM, Noyes N, Sikich L (2010) Double-blind maintenance safety and effectiveness findings from the Treatment of Early-Onset Schizophrenia Spectrum (TEOSS) study. J Am Acad Child Adolesc Psychiatry 49:583-594; quiz 632.

Firth J et al. (2019) The Lancet Psychiatry Commission: a blueprint for protecting physical health in people with mental illness. Lancet Psychiatry 6:675-712.

Green AI, Lieberman JA, Hamer RM, Glick ID, Gur RE, Kahn RS, McEvoy JP, Perkins DO, Rothschild AJ, Sharma T, Tohen MF, Woolson S, Zipursky RB (2006) Olanzapine and haloperidol in first episode psychosis: two-year data. Schizophr Res 86:234-243.

Huang M, Yu L, Pan F, Lu S, Hu S, Hu J, Chen J, Jin P, Qi H, Xu Y (2018) A randomized, 13-week study assessing the efficacy and metabolic effects of paliperidone palmitate injection and olanzapine in first-episode schizophrenia patients. Prog Neuropsychopharmacol Biol Psychiatry 81:122-130.

Huhn M, Nikolakopoulou A, Schneider-Thoma J, Krause M, Samara M, Peter N, Arndt T, Backers L, Rothe P, Cipriani A, Davis J, Salanti G, Leucht S (2019) Comparative efficacy and tolerability of 32 oral antipsychotics for the acute treatment of adults with multi-episode schizophrenia: a systematic review and network meta-analysis. Lancet 394:939-951.

Kahn RS, Fleischhacker WW, Boter H, Davidson M, Vergouwe Y, Keet IP, Gheorghe MD, Rybakowski JK, Galderisi S, Libiger J, Hummer M, Dollfus S, Lopez-Ibor JJ, Hranov LG, Gaebel W, Peuskens J, Lindefors N, Riecher-Rossler A, Grobbee DE (2008) Effectiveness of antipsychotic drugs in first-episode schizophrenia and schizophreniform disorder: an open randomised clinical trial. Lancet 371:1085-1097.

Kahn RS, Winter van Rossum I, Leucht S, McGuire P, Lewis SW, Leboyer M, Arango C, Dazzan P, Drake R, Heres S, Díaz-Caneja CM, Rujescu D, Weiser M, Galderisi S, Glenthøj B, Eijkemans MJC, Fleischhacker WW, Kapur S, Sommer IE (2018) Amisulpride and olanzapine followed by open-label treatment with clozapine in first-episode schizophrenia and schizophreniform disorder (OPTiMiSE): a three-phase switching study. Lancet Psychiatry 5:797-807.

Kishimoto T, Hagi K, Nitta M, Kane JM, Correll CU (2019) Long-term effectiveness of oral second-generation antipsychotics in patients with schizophrenia and related disorders: a systematic review and meta-analysis of direct head-to-head comparisons. World Psychiatry 18:208-224.

Larsson SC, Bäck M, Rees JMB, Mason AM, Burgess S (2020) Body mass index and body composition in relation to 14 cardiovascular conditions in UK Biobank: a Mendelian randomization study. Eur Heart J 41:221-226.

Law MR, Soumerai SB, Ross-Degnan D, Adams AS (2008) A longitudinal study of medication nonadherence and hospitalization risk in schizophrenia. J Clin Psychiatry 69:47-53.

Leucht S, Cipriani A, Spineli L, Mavridis D, Orey D, Richter F, Samara M, Barbui C, Engel RR, Geddes JR, Kissling W, Stapf MP, Lassig B, Salanti G, Davis JM (2013) Comparative efficacy and tolerability of 15 antipsychotic drugs in schizophrenia: a multiple-treatments meta-analysis. Lancet 382:951-962.

Li YM, Zhao JP, Ou JJ, Wu RR (2012) Efficacy and tolerability of ziprasidone vs. olanzapine in naive first-episode schizophrenia: a 6-week, randomized, open-label, flexible-dose study. Pharmacopsychiatry 45:177-181.

Liberati A, Altman DG, Tetzlaff J, Mulrow C, Gotzsche PC, Ioannidis JP, Clarke M, Devereaux PJ, Kleijnen J, Moher D (2009) The PRISMA statement for reporting systematic reviews and meta-analyses of studies that evaluate health care interventions: explanation and elaboration. J Clin Epidemiol 62:e1-e34.

Lieberman JA, Stroup TS, McEvoy JP, Swartz MS, Rosenheck RA, Perkins DO, Keefe RS, Davis SM, Davis CE, Lebowitz BD, Severe J, Hsiao JK (2005) Effectiveness of antipsychotic drugs in patients with chronic schizophrenia. N Engl J Med 353:1209-1223.

Lieberman JA, Tollefson G, Tohen M, Green AI, Gur RE, Kahn R, McEvoy J, Perkins D, Sharma T, Zipursky R, Wei H, Hamer RM (2003) Comparative efficacy and safety of atypical and conventional antipsychotic drugs in first-episode psychosis: a randomized, double-blind trial of olanzapine versus haloperidol. Am J Psychiatry 160:1396-1404.

Maayan L, Correll CU (2010) Management of antipsychotic-related weight gain. Expert Rev Neurother 10:1175-1200.

McEvoy JP, Lieberman JA, Perkins DO, Hamer RM, Gu H, Lazarus A, Sweitzer D, Olexy C, Weiden P, Strakowski SD (2007) Efficacy and tolerability of olanzapine, quetiapine, and risperidone in the treatment of early psychosis: a randomized, double-blind 52-week comparison. Am J Psychiatry 164:1050-1060.

Meyer JM, Davis VG, Goff DC, McEvoy JP, Nasrallah HA, Davis SM, Rosenheck RA, Daumit GL, Hsiao J, Swartz MS, Stroup TS, Lieberman JA (2008) Change in metabolic syndrome parameters with antipsychotic treatment in the CATIE Schizophrenia Trial: prospective data from phase 1. Schizophr Res 101:273-286.

Moisan J, Gregoire JP, Gaudet M, Cooper D (2005) Exploring the risk of diabetes mellitus and dyslipidemia among ambulatory users of atypical antipsychotics: a population-based comparison of risperidone and olanzapine. Pharmacoepidemiol Drug Saf 14:427-436.

Newcomer JW (2005) Second-generation (atypical) antipsychotics and metabolic effects: a comprehensive literature review. CNS Drugs 19(Suppl 1):1-93.

Ou JJ, Xu Y, Chen HH, Fan X, Gao K, Wang J, Guo XF, Wu RR, Zhao JP (2013) Comparison of metabolic effects of ziprasidone versus olanzapine treatment in patients with first-episode schizophrenia. Psychopharmacology (Berl) 225:627-635.

Patel JK, Buckley PF, Woolson S, Hamer RM, McEvoy JP, Perkins DO, Lieberman JA (2009) Metabolic profiles of second-generation antipsychotics in early psychosis: findings from the CAFE study. Schizophr Res 111:9-16.

Perez-Iglesias R, Crespo-Facorro B, Amado JA, Garcia-Unzueta MT, Ramirez-Bonilla ML, Gonzalez-Blanch C, Martinez-Garcia O, Vazquez-Barquero JL (2007) A 12-week randomized clinical trial to evaluate metabolic changes in drug-naive, first-episode psychosis patients treated with haloperidol, olanzapine, or risperidone. J Clin Psychiatry 68:1733-1740.

Perez-Iglesias R, Crespo-Facorro B, Martinez-Garcia O, Ramirez-Bonilla ML, Alvarez-Jimenez M, Pelayo-Teran JM, Garcia-Unzueta MT, Amado JA, Vazquez-Barquero JL (2008) Weight gain induced by haloperidol, risperidone and olanzapine after 1 year: findings of a randomized clinical trial in a drug-naive population. Schizophr Res 99:13-22.

Perez-Iglesias R, Mata I, Pelayo-Teran JM, Amado JA, Garcia-Unzueta MT, Berja A, Martinez-Garcia O, Vazquez-Barquero JL, Crespo-Facorro B (2009) Glucose and lipid disturbances after 1 year of antipsychotic treatment in a drug-naive population. Schizophr Res 107:115-121.

Pillinger T, McCutcheon RA, Vano L, Mizuno Y, Arumuham A, Hindley G, Beck K, Natesan S, Efthimiou O, Cipriani A, Howes OD (2020) Comparative effects of 18 antipsychotics on metabolic function in patients with schizophrenia, predictors of metabolic dysregulation, and association with psychopathology: a systematic review and network meta-analysis. Lancet Psychiatry 7:64-77.

Poyurovsky M, Pashinian A, Gil-Ad I, Maayan R, Schneidman M, Fuchs C, Weizman A (2002) Olanzapine-induced weight gain in patients with first-episode schizophrenia: a double-blind, placebo-controlled study of fluoxetine addition. Am J Psychiatry 159:1058-1060.

R Foundation. Previous releases of R for Windows. CRAN R Project, 2022. Available at: https://cran.r-project.org/bin/windows/base/old/. Accessed: June 23, 2022.

Raben AT, Marshe VS, Chintoh A, Gorbovskaya I, Muller DJ, Hahn MK (2018) The complex relationship between antipsychotic-induced weight gain and therapeutic benefits: a systematic review and implications for treatment. Front Neurosci 11:741.

Saddichha S, Ameen S, Akhtar S (2008a) Predictors of antipsychotic-induced weight gain in first-episode psychosis: conclusions from a randomized, double-blind, controlled prospective study of olanzapine, risperidone, and haloperidol. J Clin Psychopharmacol 28:27-31.

Saddichha S, Manjunatha N, Ameen S, Akhtar S (2008b) Diabetes and schizophrenia—effect of disease or drug? Results from a randomized, double-blind, controlled prospective study in first-episode schizophrenia. Acta Psychiatr Scand 117:342-347.

Saddichha S, Manjunatha N, Ameen S, Akhtar S (2008c) Metabolic syndrome in first episode schizophrenia—a randomized double-blind controlled, short-term prospective study. Schizophr Res 101:266-272.

San L, Arranz B, Perez V, Safont G, Corripio I, Ramirez N, Dueñas R, Alvarez E (2012) One-year, randomized, open trial comparing olanzapine, quetiapine, risperidone and ziprasidone effectiveness in antipsychotic-naive patients with a first-episode psychosis. Psychiatry Res 200:693-701.

Sanger TM, Lieberman JA, Tohen M, Grundy S, Beasley C, Jr., Tollefson GD (1999) Olanzapine versus haloperidol treatment in first-episode psychosis. Am J Psychiatry 156:79-87.

Sikich L, Frazier JA, McClellan J, Findling RL, Vitiello B, Ritz L, Ambler D, Puglia M, Maloney AE, Michael E, De Jong S, Slifka K, Noyes N, Hlastala S, Pierson L, McNamara NK, Delporto-Bedoya D, Anderson R, Hamer RM, Lieberman JA (2008) Double-blind comparison of first- and second-generation antipsychotics in early-onset schizophrenia and schizo-affective disorder: findings from the Treatment of Early-Onset Schizophrenia Spectrum Disorders (TEOSS) Study. Am J Psychiatry 165:1420-1431.

Solmi M, Murru A, Pacchiarotti I, Undurraga J, Veronese N, Fornaro M, Stubbs B, Monaco F, Vieta E, Seeman MV, Correll CU, Carvalho AF (2017) Safety, tolerability, and risks associated with first- and second-generation antipsychotics: a state-of-the-art clinical review. Ther Clin Risk Manag 13:757-777.

Sterne JAC et al. (2019) RoB 2: a revised tool for assessing risk of bias in randomised trials. BMJ 366:l4898.

Toledo FGS, Martin WF, Morrow L, Beysen C, Bajorunas D, Jiang Y, Silverman BL, McDonnell D, Namchuk MN, Newcomer JW, Graham C (2022) Insulin and glucose metabolism with olanzapine and a combination of olanzapine and samidorphan: exploratory phase 1 results in healthy volunteers. Neuropsychopharmacology 47:696-703.

Vancampfort D, Stubbs B, Mitchell AJ, De Hert M, Wampers M, Ward PB, Rosenbaum S, Correll CU (2015) Risk of metabolic syndrome and its components in people with schizophrenia and related psychotic disorders, bipolar disorder and major depressive disorder: a systematic review and meta-analysis. World Psychiatry 14:339-347.

Wu RR, Zhao JP, Liu ZN, Zhai JG, Guo XF, Guo WB, Tang JS (2006) Effects of typical and atypical antipsychotics on glucose-insulin homeostasis and lipid metabolism in first-episode schizophrenia. Psychopharmacology (Berl) 186:572-578.

Wu RR, Zhao JP, Zhai JG, Guo XF, Guo WB (2007) Sex difference in effects of typical and atypical antipsychotics on glucose-insulin homeostasis and lipid metabolism in first-episode schizophrenia. J Clin Psychopharmacol 27:374-379.

Yildiz A, Nikodem M, Vieta E, Correll CU, Baldessarini RJ (2015) A network meta-analysis on comparative efficacy and all-cause discontinuation of antimanic treatments in acute bipolar mania. Psychol Med 45:299-317.

Zhang S, Lan G (2014) Prospective 8-week trial on the effect of olanzapine, quetiapine, and aripiprazole on blood glucose and lipids among individuals with first-onset schizophrenia. Shanghai Arch Psychiatry 26:339-346.

Zheng Y, Manson JE, Yuan C, Liang MH, Grodstein F, Stampfer MJ, Willett WC, Hu FB (2017) Associations of weight gain from early to middle adulthood with major health outcomes later in life. JAMA 318:255-269.

Zhu Y, Krause M, Huhn M, Rothe P, Schneider-Thoma J, Chaimani A, Li C, Davis JM, Leucht S (2017) Antipsychotic drugs for the acute treatment of patients with a first episode of schizophrenia: a systematic review with pairwise and network meta-analyses. The lancet Psychiatry 4:694-705.

Zipursky RB, Gu H, Green AI, Perkins DO, Tohen MF, McEvoy JP, Strakowski SM, Sharma T, Kahn RS, Gur RE, Tollefson GD, Lieberman JA (2005) Course and predictors of weight gain in people with first-episode psychosis treated with olanzapine or haloperidol. Br J Psychiatry 187:537-543.
